# A case control study of differences in non-work injury and accidents among sawmill workers in rural compared to urban British Columbia, Canada

**DOI:** 10.1186/1471-2458-9-432

**Published:** 2009-11-25

**Authors:** Aleck Ostry, Stefania Maggi, Ruth Hershler, Lisa Chen, Amber Louie, Clyde Hertzman

**Affiliations:** 1 Department of Geography, PO BOX 3060 STN CSC, University of Victoria, Victoria, B.C., V8W 3R4, Canada; 2 Department of Psychology and Institute of Interdisciplinary Studies, Dunton Tower Room 2210, Carleton University, 1125 Colonel By Drive, Ottawa, ON K1S 5B6, Canada; 3 Human Early Learning Program, University of British Columbia, 4th Floor, Library Processing Centre, 2206 East Mall, Vancouver, B.C., V6T 1Z3, Canada

## Abstract

**Background:**

Using a cohort of British Columbian male sawmill workers, we conducted a nested case-control study of the impact of rural compared to urban residence as well as rural/urban migration patterns in relation to hospitalization for non-work injury. We postulate that for many types of non-work injuries, rates will be higher in rural communities than in urban ones and that rates will also be higher for workers who migrate from urban to rural communities.

**Methods:**

Using conditional logistic regression, univariate models were first run with each of five non-work injury outcomes. These outcomes were hospitalizations due to assault, accidental poisoning, medical mis-adventure, motor vehicle trauma, and other non-work injuries. In multivariate models marital status, ethnicity, duration of employment, and occupation were forced into the model and associations with urban, compared to rural, residence and various urban/migration patterns were tested.

**Results:**

Urban or rural residence and migration status from urban to other communities, and across rural communities, were not associated with hospitalization for medical misadventure, assault, or accidental poisoning. The likelihood of a rural resident being hospitalized for motor vehicle trauma is higher than for an urban resident. The likelihood that a rural resident is hospitalized for "other" non-work injury is higher than for an urban resident.

**Conclusion:**

In a relatively homogenous group of workers, and using a rigorous study design, we have demonstrated that the odds of other non-work injury are much higher for workers resident in and migrating to rural regions of Canada than they are for workers resident in or migrating to urban places.

## Background

According to an investigation into rural health conducted by the Canadian Population Health Initiative, the health status of rural Canadians is systematically worse than it is for urban Canadians, for most, but not all, outcomes [1,2]. This report demonstrated that urban-rural differences in health status, in morbidity and mortality from most illnesses, and health behaviours remain even after controlling for socio-economic status, suggesting that something about rural life in itself accounts for these differences.

The report also clearly illustrated the major differences in socio-economic status between urban and rural Canadians. For example, in remote areas of Canada approximately 50 percent of the population has little formal education compared to a figure of approximately 25 percent in urban regions. This underscores the fact that any analyses comparing health outcomes between residents in rural and urban areas must take into account the large differences in labour market, income, and educational status. When investigating health outcomes across the rural/urban continuum, it is important to be able to control for confounding by various measures of socio-economic status and/or conduct studies among sub-populations that are similar across regions.

Non-work injury, except perhaps in the case of motor vehicle trauma, remains under-investigated in studies of rural health. There is evidence that in the case of motor vehicle trauma, that in North America, both injuries and fatalities occur in higher proportion and with greater severity in rural compared to urban areas [3,4]. For non-work injury outcomes such as assault, medical misadventure, and accidental poisoning, there is little understanding of differences across rural and urban places.

In recent years a growing body of evidence has been generated investigating occupational and community influences on a wide range of health outcomes using a large cohort of British Columbian sawmill workers and their children [5-16]. The present study explores differences between urban and rural sawmill worker members of this cohort in hospitalization for non-work injury.

Because the nature and organization of work in sawmills located in rural and urban regions of the province is fairly similar and because educational requirements and wages paid have been similar throughout the province, the cohort members are a homogeneous group. As well, there is information, for cohort members, on potential socio-economic confounders. Using this cohort in investigations of rural/urban differences in health, provides a unique opportunity to compare health outcomes within a relatively homogeneous population of workers for which variables are available to further control for socio-economic confounding.

The aim of the study was to explore the relationship between rural and urban residency and migration between rural and urban places and the risk of hospitalization for non-work related injury among BC sawmill workers

### Literature Review

This literature review is divided into four sections. In the first section we review the Canadian literature on rural/urban differences in motor vehicle and other vehicular accidents. Because there is no available Canadian research on rural/urban differences in accidental poisonings and non-work injuries, in the second section we review the, although limited, international literature on this topic. In the third section we review the limited Canadian literature on rural/urban differences in assault. Finally, in the fourth section, we review the international literature on rural/urban differences in medical misadventure.

### Motor Vehicle Trauma

Across North America injuries and fatalities due to motor vehicle trauma occur in higher proportion and greater severity in rural compared to urban areas [3,4]. According to Transport Canada in 2002, 63.2% of all fatal crashes occurred on rural roadways [17]. In Alberta in 2002, nearly 75% of fatal crashes occurred in rural areas [17-19]. These studies also illustrated that speeding and not wearing seat belts were more prevalent in rural than in urban areas.

In Alberta, rural residents and men were more likely to sustain spinal injuries, mainly due to vehicle accidents [20]. Using data from the Canadian Institute for Health Information (CIHI), Macpherson et al. [19] investigated all Canadian children hospitalized due to bicycling-related injuries (1994-1998, n = 9367). The average annual incidence rate for bicycle-related head injuries in children was 18.5 per 100, 000 for children living in rural compared with 10.9 in urban areas, 15.5 in mixed urban and 17.4 in mixed rural areas. Logistic regression, controlling for age, sex, socio-economic status (SES), collision with a motor vehicle, and the presence of provincial helmet legislation, suggested that this variation may be explained by differences in bicycling exposure, helmet use, hospital admission criteria, or road environments across geographic areas.

A population-based study of motor vehicle trauma among children and youth in Alberta examined police report data for the period from 1997 to 2002 [21]. Across all age and sex strata, both hospitalization and fatality rates were significantly higher in rural compared with urban regions. After adjusting for age, sex, and calendar year, the relative risk for motor vehicle trauma hospitalization (rural versus urban) was 3.0 (95% CI: 2.8, 3.2), and for fatality, 5.4 (95% CI: 4.2, 6.9).

### Accidental poisoning and Other non-work injuries

The research on rural/urban differences in accidental poisoning and non-work injuries in Canada is non-existent. However, a limited number of researchers in other countries have examined this topic. Boland and colleagues [22] conducted a study of urban/rural differences in mortality and hospital admission rates for non-work injuries in the Republic of Ireland. Central Statistics Office mortality data from 1980-2000 were used to calculate standardized mortality ratios (SMRs) in residents of urban and rural areas, and standardized hospital admission ratios (SARs) in urban and rural residents were calculated using hospital admission data (Hospital In-Patient Enquiry) from 1993-2000. The overall rate of non-work injury mortality was significantly higher among rural residents (SMR 103.0, 95% CI 101-105), and also for deaths related to drowning, accidents and injury from machinery, and firearms. Among rural residents SARs were significantly higher for injuries from falls, being struck by or against an object, fire or burns, and accident and injury from machinery.

A cross-sectional study of poisoning of children aged 0-4 years based on crude rates of hospitalizations in Australia during the financial year 1996-97 found significantly higher rates among children living in rural and remote areas compared with those living in metropolitan areas [23]. Rate differentials increased with geographical remoteness.

A UK study calculated SMRs using data from the longitudinal study of the Office of Population Censuses and Surveys, a quasi-random 1% sample of the population of England and Wales [24]. In general, the results demonstrated a striking similarity between metropolitan and non-metropolitan areas, for deaths from accident, violence, and poison.

### Assault

A 1991 Canadian study on domestic homicide involving firearms [25] showed that almost half (49%) of domestic homicides occurred in rural areas (i.e., places with a population less than 10,000), even though rural residences account for only 23% of the population. However, consistent with previous research, urban dwellers report higher rates of personal victimization--including sexual assault, robbery, assault, break and enter, motor vehicle/parts theft and vandalism--than those from rural areas. Urban residents reported a total personal victimization rate over 40% higher than that of rural dwellers (199 versus 138 per 1,000) [26].

Estimates of the rates of violence against women in rural Canada are few [27]. The Statistic Canada General Social Survey found no variation in reported rates of spousal violence between urban and rural men and women [28]. However, in rural areas, 2% of women and 1% of men reported spousal violence in the past 12 months by their current partners, compared to 1% of women and 2% of men in urban areas. Notably though, availability of services that address domestic violence, including health services, is lower in rural areas. This may reduce reporting rates for rural citizens [27]. In a cross-sectional survey in a rural health region in Alberta, among 526 women, 5% of women reported experiencing physical assault in the last 12 months and 23% reported experiencing sexual assault in their lifetime, indicating that rates of spousal abuse in rural regions are moderately high [29].

### Medical Misadventure

There is a lack of research on how patient safety and quality of care differ between rural and urban settings [30]. A review of the limited available research, mainly from the United States, suggests that patient safety events and medical errors may be less likely to occur in rural than in urban hospitals [31]. For example, Romano et al. [32] conducted a study of patient safety in the U.S. [33]. They found that the incidence of most Patient Safety Incidents was highest at urban teaching hospitals. The Harvard Medical Practice Study, conducted in acute care hospitals in New York State in 1984 also showed significantly lower medical injury rates in rural compared to metropolitan hospitals [34].

## Methods

Using all the International Classification of Diseases E-codes available from the BC Linked Health Database (BCLHDB) [35] we were able to study rural/urban differences in hospitalization for the following five outcomes: 1) assault; 2) accidental poisonings; 3) medical misadventures; 4) motor vehicle trauma; and 5) other non-work injuries. These E-codes pertain only to non-work injury. As well, because we have information on migration patterns and not just current place of residence, we were able to assess the influence of different migration patterns between urban and rural, as well as across rural communities.

This study is based on a cohort of male sawmill workers which was assembled in the 1980s to assess the effects of chemical exposure on mortality and cancer among BC sawmill workers; it has been adapted for use in this present investigation. In the original study, fourteen large sawmills (150 to 450 workers each) were identified, some in urban and others in rural areas. The personnel records of workers employed for at least one year between 1950 and 1998 were used to identify the study participants. This provided us with detailed personal and job history information for 28,794 workers employed at these mills from 1950 through to 1998 [7].

### Definition of Rural and Urban Utilized in the Present Study

For our definition of rural and urban we determined if the population of each place where the workers were diagnosed was greater or less than 100,000. If it was less than 100,000 the place of diagnosis was classified as rural, and if it was over 100,000 it was urban.

### Obtaining Information on Non-Work Injury Outcomes

Health information for each cohort member was obtained by probabilistic linkage to the BC Linked Health Database (BCLHDB) which has files on physician services utilization and hospital discharges from 1985 to the present. The records are housed at the University of British Columbia's Population Health Observatory. The BCLHDB is managed according to the provisions of British Columbia's Freedom on Information and Protection of Privacy Act. Ethical approval to conduct this study was obtained from the University of British Columbia (UBC) and the British Columbia Ministry of Health

There are approximately 120 "E" codes characterizing a range of non-work accidents and injuries in the hospital discharge data. We utilized approximately 100 of these codes (See Additional File 1) in order to develop five general categories of non-work injury and accident for the purposes of this investigation. The five categories are as follows: 1) assault; 2) accidental poisonings; 3) medical misadventures; 4) motor vehicle trauma; 5) other non-work injuries and accidents. Approximately 20 codes did not fit in these 5 basic categories and were thus excluded from analysis.

### Selection of Cases and Controls

There were several reasons for using a nested case control design. First, we were able to determine non-work injury outcomes across several major diagnostic categories. Second, the study statistically controlled for residual confounding by socio-economic factors, thus increasing comparability between workers living in urban and rural settings. Third, the study was longitudinal in design and utilized common International Classification of Disease (ICD 9) codes for non-work injury outcomes, based upon a common data source for urban and rural study subjects. Finally, because of its historical prospective character we were able to address confounding by migration, identifying workers who migrated between rural and urban environments before and after diagnosis.

Complete hospital diagnoses for these five categories of non-work accidents and injury were available in the BCLHDB [35] from January 1st 1994 until December 31st, 2001. Cases were eligible for selection from this 8-year period. Cases included all subjects with a first ICD9 diagnostic code for these five categories. We identified 151 hospital discharges for assault, 75 cases of accidental poisonings, 1,073 cases of medical misadventure, 470 cases for motor vehicle trauma, and 2,046 cases of other non-work injury.

For each case we identified the place they were living when diagnosed with a non-work related injury, using postal codes available in the BCLHDB. In this way we were able to determine whether a case that originated at an urban mill had remained in their same urban location (urban stay), or had moved away from this mill (urban migrate). Similarly, we determined whether a case that originated at a rural mill remained at the same location (rural stay), moved to an urban location (rural urban), or moved to another rural location (rural rural). This classification scheme therefore identified two types of cases that were non-migrators (those who stayed in the same urban location and those who remained in the same rural location), as well as three types of cases involving migration (those urban dwellers who migrated away from their original urban location, rural dwellers who migrated to an urban place, and rural dwellers who migrated away from their original rural place to another rural place). Note that we did not determine whether the urban dwellers who migrated away from their original urban location moved to another urban place or to a rural one, only that they migrated away from an urban location.

Using STTOCC (survival-time to case-control) on STATA 8.0, three controls were selected for each case matched on age. Controls were chosen randomly with replacement from the set at risk, that is, all the members of the cohort who worked in a study sawmill for at least one year. Thus, a control could be anyone at risk who also satisfied the matching criteria, and who had not had a non-work related injury up to the time of diagnosis of the case.

## Results

No significant associations were observed in univariate analyses for assault, accidental poisoning, or medical misadventure. Univariate analyses indicate that those who stayed in urban regions had a lower Odds Ratio for motor vehicle trauma (.60; CI .42-.86), and those who migrated from rural to a different rural area had elevated Odds Ratios for motor vehicle trauma (1.49; CI 1.19-1.86). In multivariate models, after controlling for socio-demographic variables, duration of employment, and occupation, workers who migrated from one rural community to another had approximately twice the odds of motor vehicle trauma than workers who remained in an urban community. Statistically significant and elevated rates were also observed for rural compared to urban residents (OR = 1.56; CI 1.01-2.40), and for workers who moved from a rural to urban community (OR = 1.76; CI 1.11-2.79). As well, in this model, relative to managers, skilled workers were approximately 2.5 times as likely, and unskilled workers twice as likely, to sustain a motor vehicle trauma (Tables 1, 2).

**Table 1:** Univariate analyses: Odds ratios for motor vehicle trauma and other non-work injury among sawmill workers for the period 1994 through 2001

	Motor Vehicle Trauma (N = 470)	Other non-work injury (N = 2,046)
Urban stay	.60 (.42, .86).	.65 (.53, .80)
Urban migrate	.79 (.60, 1.04)	.96 (.82, 1.13)
Rural stay	1.01 (.76, 1.35)	1.43 (1.18, 1.74)
Rural to urban	1.15 (.86, 1.54)	.82 (.67, .99)
Rural to rural	1.49 (1.19, 1.86)	1.33 (1.16, 1.53)

* Numbers in parentheses are 95% Confidence Intervals.

**Table 2:** Multivariate analyses: Odds ratios for motor vehicle trauma and other non-work injury among sawmill workers for the period 1994 through 2001

	Motor Vehicle Trauma N = 470	Other non-Work Injury N = 2,046
Duration of job at		
sawmill (years)	.99 (.97, 1.01)	.99 (.98, 1.01)
Marital status	1.02 (0.97, 1.07)	1.02 (.99, 1.05)
Tradesman	1.99 (.98, 4.04)	1.04(.72, 1.45)
Skilled	2.49 (1.20, 5.17)	.95 (.66, 1.35)
Unskilled	2.05 (.1.01, 4.17)	.96 (.68, 1.34)
Caucasian	1	1
Chinese	1.07 (.34, 3.35)	.23 (.08, .70)
Sikh	1.30 (.86, 1.96)	.84 (.61, 1.16)
Urban stay	1	1
Urban migrate	1.35 (.87, .2.09)	1.34 (1.04, 1.72)
Rural stay	1.56 (1.01, 2.40)	1.94 (1.49, 2.53)
Rural to urban	1.76 (1.11, 2.79)	1.12 (.85, 1.48)
Rural to rural	2.09 (1.40, 3.13)	1.63 (1.29, 2.07)

* Numbers in parentheses are 95% Confidence Intervals.

Univariate analyses for other non-work injury indicate reduced odds for urban stayers (OR = 0.65; CI .53-.80) and for those workers who migrated from rural to urban communities (OR = 0.82; CI .67-.99). In contrast elevated odds were observed for rural stayers (OR = 1.43; CI 1.18-1.74) and those who migrated from rural to another rural community (OR = 1.33; CI 1.16-1.53). Multivariate models, after controlling for socio-demographic variables, duration of employment, and occupation, showed even higher odds for rural stayers (OR = 1.94; CI 1.49-2.53) and for migrants from rural to other rural communities (OR = 1.63; CI 1.29-2.07). Additionally, these multivariate models showed elevated odds (OR = 1.34; CI 1.04-1.72) for workers who migrated away from urban communities (Tables 1 &2).

## Discussion

There are three main results from this study. First, urban or rural residence and migration status from urban to other communities, and across rural communities, was not associated with hospitalization for assault, accidental poisoning, or medical misadventure. The results for assault accord with the limited literature on rural/urban differences in Canada.

Second, in accord with existing Canadian research, the likelihood of a rural resident being hospitalized for motor vehicle trauma is higher than for an urban resident. Our research indicates that, relative to urban dwellers, rates are even higher for cohort members who move from one rural community to another rural community. As well, workers who migrate from urban communities have a greater likelihood of being involved in a motor vehicle trauma. We did not track the types of communities that these urban migrators moved to, it may be that most moved to rural communities, however in this study their destination remains unknown.

Third, in accord with the limited Canadian research, the likelihood that a rural resident is hospitalized for other non-work injury is higher than for an urban resident. Although the odds are somewhat lower for workers who migrate from one rural community to another, they are still higher than for workers who remain in urban communities.

Even among a group of workers employed in the same type of industry, and even after strict controls for confounding related to demographic and occupational factors, the odds for motor vehicle trauma and other non-work injury are significantly higher for rural workers compared to their urban counterparts. This finding points to structural features of rural non-work and recreational life and activity as risks for greater hospitalization for these two outcomes. Road safety in rural areas is a major health issue, and improvements in this regard may help to redress some of the imbalance in motor vehicle trauma outcomes for rural residents. As well, safety in outdoor rural recreational pursuits and in non-paid work pursuits such as do-it-yourself home renovations may pay off in terms of reducing hospitalization for these outcomes.

There are several limitations to this study. Outcomes were ICD 9 codes based on hospitalized cases. They do not capture less severe non-work injury cases. As well, this study is based on males only. As it is an unusual population of workers this study is not representative of the general population and so the findings cannot be generalized. The definition of rural used in this investigation is very broad; rural place was defined simply as any population center with less than 100,000 people. So, in effect we are measuring the difference between residents of Census Metropolitan Areas (CMAs) versus "elsewhere." This threshold for rurality is much higher than is used in most other urban/rural investigations, and so limits the comparability of this study to others.

Finally, our classification of workers migratory trajectories was crude. In particular, we did not divide urban migrators into those who migrate to other urban places and those who migrate from urban to rural places. However, despite these limitations there are a great many strengths to this study.

The study was rigorous in design utilizing objective measures of non-work injury, fine control for socio-economic differences among participants, and it was longitudinal. Furthermore, this study is based on a population that was selected based on its employment status, so it largely excluded unhealthy people. Finally, as most researchers on the rural/urban health divide have noted, it is important in studies of this type to measure not only non-work injury outcomes among rural and urban residents but also to assess outcomes among migrants; this study does exactly that.

## Conclusion

In a relatively homogenous group of workers, and using a rigorous study design, we have demonstrated that the odds of other non-work injury are much higher for workers resident in and migrating to rural regions of Canada than they are for workers resident in or migrating to urban places.

## Competing interests

The authors declare that they have no competing interests.

## Authors' contributions

AO was PI on the study to obtain funding for this research, directed the analysis, and was the lead writer. SM took a lead on the analysis and reviewed drafts. RH conducted the analysis and LC managed the database. AL assisted with the literature review. CH conducted the research, helped direct the analysis, and read drafts of the paper.

All authors read and approved the final manuscript.

## Pre-publication history

The pre-publication history for this paper can be accessed here:

http://www.biomedcentral.com/1471-2458/9/432/prepub

## Supplementary Material

Additional file 1 **"E-Codes" available in BCLHDB Hospital Discharge Records and Collapsed into Five Major Categories**. This is a table outlining how the E codes for injury were aggregated into five categories. Click here for file
