# Supplementary material for: A case control study of differences in non-work injury and accidents among sawmill workers in rural compared to urban British Columbia, Canada
Source: BMC Public Health. 2009 Nov 25;9:432. doi: 10.1186/1471-2458-9-432 (PMC2789069; doi:10.1186/1471-2458-9-432)
Supplement: Additional file 1 — "E-Codes" available in BCLHDB Hospital Discharge Records and Collapsed into Five Major Categories. This is a table outlining how the E codes for injury were aggregated into five categories. [file 1471-2458-9-432-S1.DOCX]

**“E-Codes” available in BCLHDB Hospital Discharge Records and Collapsed into Five Major Categories**

| **Group 1 - Assaults** |  |
| --- | --- |
| ASSAULT BY FIREARMS AND EXPLOSIVES | |
| ASSAULT BY CUTTING AND PIERCING INSTRUMENT | |
| ASSAULT BY OTHER AND UNSPECIFIED MEANS | |
| LATE EFFECTS OF INJURY PURPOSELY INFLICTED BY OTHER PERSON | |
| OTHER MOTOR VEHICLE TRAFFIC ACCIDENT INVOLVING COLLISION ON THE HIGHWAY | |
| MOTOR VEHICLE TRAFFIC ACCIDENT DUE TO LOSS OF CONTROL, WITHOUT | |
| NONCOLLISION MOTOR VEHICLE TRAFFIC ACCIDENT WHILE BOARDING OR ALIGHTING | |
| OTHER NONCOLLISION MOTOR VEHICLE TRAFFIC ACCIDENT | |
| MOTOR VEHICLE TRAFFIC ACCIDENT OF UNSPECIFIED NATURE | |
| NONTRAFFIC ACCIDENT INVOLVING MOTOR-DRIVEN SNOW VEHICLE | |
| NONTRAFFIC ACCIDENT INVOLVING OTHER OFF-ROAD MOTOR VEHICLE | |
| OTHER MOTOR VEHICLE NONTRAFFIC ACCIDENT INVOLVING COLLISION | |
| OTHER MOTOR VEHICLE NONTRAFFIC ACCIDENT INVOLVING COLLISION | |
| OTHER MOTOR VEHICLE NONTRAFFIC ACCIDENT WHILE BOARDING AND ALIGHTING | |
| OTHER MOTOR VEHICLE NONTRAFFIC ACCIDENT OF OTHER AND UNSPECIFIED NATURE | |
| PEDAL CYCLE ACCIDENT | |
| ACCIDENT INVOLVING ANIMAL BEING RIDDEN | |
| OTHER ROAD VEHICLE ACCIDENTS | |
| **Group 2 -Accidental poisonings** |  |
| ACCIDENTAL POISONING BY ANALGESICS, ANTIPYRETICS, ANTIRHEUMATICS | |
| ACCIDENTAL POISONING BY BARBITURATES | |
| ACCIDENTAL POISONING BY OTHER SEDATIVES AND HYPNOTICS | |
| ACCIDENTAL POISONING BY OTHER TRANQUILLIZERS | |
| ACCIDENTAL POISONING BY OTHER PSYCHOTROPIC AGENTS | |
| ACCIDENTAL POISONING BY OTHER DRUGS ACTING ON CENTRAL AND AUTONOMIC | |
| ACCIDENTAL POISONING BY ANTIBIOTICS | |
| ACCIDENTAL POISONING BY OTHER DRUGS | |
| ACCIDENTAL POISONING BY ALCOHOL, NOT ELSEWHERE CLASSIFIED | |
| ACCIDENTAL POISONING BY AGRICULTURAL AND HORTICULTURAL CHEMICAL AND | |
| ACCIDENTAL POISONING BY CORROSIVES AND CAUSTICS, NOT ELSEWHERE CLASSIFIE | |
| ACCIDENTAL POISONING FROM FOODSTUFFS AND POISONOUS PLANTS | |
| ACCIDENTAL POISONING BY OTHER AND UNSPECIFIED SOLID AND LIQUID SUBSTANCE | |
| ACCIDENTAL POISONING BY OTHER UTILITY GAS AND OTHER CARBON MONOXIDE | |
| ACCIDENTAL POISONING BY OTHER GASES AND VAPOURS | |
| POISONING BY SOLID OR LIQUID SUBSTANCES, UNDETERMINED WHETHER | |
| POISONING BY OTHER GASES, UNDETERMINED WHETHER ACCIDENTALLY OR | |
| **Group 3 - Medical Misadventures** |  |
| SURGICAL OPERATION AND OTHER SURGICAL PROCEDURES AS THE CAUSE OF | |
| OTHER PROCEDURES, WITHOUT MENTION OF MISADVENTURE AT THE TIME OF | |
| ACCIDENTAL CUT, PUNCTURE, PERFORATION OR HAEMORRHAGE DURING MEDICAL CARE | |
| FOREIGN OBJECT LEFT IN BODY DURING PROCEDURE | |
| FAILURE OF STERILE PRECAUTIONS DURING PROCEDURE | |
| MECHANICAL FAILURE OF INSTRUMENT OR APPARATUS DURING PROCEDURE | |
| **Group 4 - Injuries involving vehicles** | |
| MOTOR VEHICLE TRAFFIC ACCIDENT INVOLVING RE-ENTRANT COLLISION WITH | |
| OTHER MOTOR VEHICLE TRAFFIC ACCIDENT INVOLVING COLLISION WITH ANOTHER | |
| MOTOR VEHICLE TRAFFIC ACCIDENT INVOLVING COLLISION WITH OTHER VEHICLE | |
| MOTOR VEHICLE TRAFFIC ACCIDENT INVOLVING COLLISION WITH PEDESTRIAN | |
| OTHER MOTOR VEHICLE TRAFFIC ACCIDENT INVOLVING COLLISION ON THE HIGHWAY | |
| MOTOR VEHICLE TRAFFIC ACCIDENT DUE TO LOSS OF CONTROL, WITHOUT | |
| NONCOLLISION MOTOR VEHICLE TRAFFIC ACCIDENT WHILE BOARDING OR ALIGHTING | |
| OTHER NONCOLLISION MOTOR VEHICLE TRAFFIC ACCIDENT | |
| MOTOR VEHICLE TRAFFIC ACCIDENT OF UNSPECIFIED NATURE | |
| NONTRAFFIC ACCIDENT INVOLVING MOTOR-DRIVEN SNOW VEHICLE | |
| NONTRAFFIC ACCIDENT INVOLVING OTHER OFF-ROAD MOTOR VEHICLE | |
| OTHER MOTOR VEHICLE NONTRAFFIC ACCIDENT INVOLVING COLLISION | |
| OTHER MOTOR VEHICLE NONTRAFFIC ACCIDENT INVOLVING COLLISION | |
| OTHER MOTOR VEHICLE NONTRAFFIC ACCIDENT WHILE BOARDING AND ALIGHTING | |
| OTHER MOTOR VEHICLE NONTRAFFIC ACCIDENT OF OTHER AND UNSPECIFIED NATURE | |
| PEDAL CYCLE ACCIDENT | |
| ACCIDENT INVOLVING ANIMAL BEING RIDDEN | |
| **Group 5 – Other Non-work Injuries** | |
| FALL ON OR FROM STAIRS OR STEPS | |
| FALL ON OR FROM LADDERS OR SCAFFOLDING | |
| FALL FROM OR OUT OF BUILDING OR OTHER STRUCTURE | |
| FALL INTO HOLE OR OTHER OPENING IN SURFACE | |
| OTHER FALL FROM ONE LEVEL TO ANOTHER | |
| FALL ON SAME LEVEL FROM SLIPPING, TRIPPING OR STUMBLING | |
| FALL ON SAME LEVEL FROM COLLISION, PUSHING OR SHOVING, BY OR WITH OTHER | |
| FRACTURE, CAUSE UNSPECIFIED | |
| OTHER AND UNSPECIFIED FALL | |
| CONFLAGRATION IN PRIVATE DWELLING | |
| CONFLAGRATION IN OTHER AND UNSPECIFIED BUILDING OR STRUCTURE | |
| ACCIDENT CAUSED BY IGNITION OF CLOTHING | |
| IGNITION OF HIGHLY INFLAMMABLE MATERIAL | |
| ACCIDENT CAUSED BY CONTROLLED FIRE IN PRIVATE DWELLING | |
| ACCIDENT CAUSED BY CONTROLLED FIRE NOT IN BUILDING OR STRUCTURE | |
| ACCIDENT CAUSED BY OTHER SPECIFIED FIRE AND FLAMES | |
| ACCIDENT CAUSED BY UNSPECIFIED FIRE | |
| EXCESSIVE HEAT | |
| EXCESSIVE COLD | |
| HUNGER, THIRST, EXPOSURE, NEGLECT | |
| VENOMOUS ANIMALS AND PLANTS AS THE CAUSE OF POISONING AND TOXIC REACTION | |
| OTHER INJURY CAUSED BY ANIMALS | |
| ACCIDENTAL DROWNING AND SUBMERSION | |
| INHALATION AND INGESTION OF FOOD CAUSING OBSTRUCTION OF RESPIRATORY | |
| INHALATION AND INGESTION OF OTHER OBJECT CAUSING OBSTRUCTION OF | |
| FOREIGN BODY ACCIDENTALLY ENTERING EYE AND ADNEXA | |
| FOREIGN BODY ACCIDENTALLY ENTERING OTHER ORIFICE | |
| STRUCK ACCIDENTALLY BY FALLING OBJECT | |
| STRIKING AGAINST OR STRUCK ACCIDENTALLY BY OBJECTS OR PERSONS | |
| CAUGHT ACCIDENTALLY IN OR BETWEEN OBJECTS | |
| ACCIDENTS CAUSED BY MACHINERY | |
| ACCIDENTS CAUSED BY CUTTING AND PIERCING INSTRUMENTS OR OBJECTS | |
| ACCIDENT CAUSED BY EXPLOSION OF PRESSURE VESSEL | |
| ACCIDENT CAUSED BY FIREARM MISSILE | |
| ACCIDENT CAUSED BY EXPLOSIVE MATERIAL | |
| ACCIDENT CAUSED BY HOT SUBSTANCE OR OBJECT, CAUSTIC OR CORROSIVE | |
| ACCIDENT CAUSED BY ELECTRIC CURRENT | |
| OVEREXERTION AND STRENUOUS MOVEMENTS | |
| OTHER AND UNSPECIFIED ENVIRONMENTAL AND ACCIDENTAL CAUSES | |
| LATE EFFECTS OF ACCIDENTAL INJURY | |
| ANTIBIOTICS | |
| OTHER ANTI-INFECTIVES | |
| HORMONES AND SYNTHETIC SUBSTITUTES | |
| PRIMARILY SYSTEMIC AGENTS | |
| AGENTS PRIMARILY AFFECTING BLOOD CONSTITUENTS | |
| ANALGESICS, ANTIPYRETICS AND ANTIRHEUMATICS | |
| ANTICONVULSANTS AND ANTI-PARKINSONISM DRUGS | |
| SEDATIVES AND HYPNOTICS | |
| OTHER CENTRAL NERVOUS SYSTEM DEPRESSANTS | |
| PSYCHOTROPIC AGENTS | |
| DRUGS PRIMARILY AFFECTING THE AUTONOMIC NERVOUS SYSTEM | |
| AGENTS PRIMARILY AFFECTING THE CARDIOVASCULAR SYSTEM | |
| AGENTS PRIMARILY AFFECTING GASTROINTESTINAL SYSTEM | |
| WATER, MINERAL AND URIC ACID METABOLISM DRUGS | |
| AGENTS PRIMARILY ACTING ON THE SMOOTH AND SKELETAL MUSCLES | |
| AGENTS PRIMARILY AFFECTING SKIN AND MUCOUS MEMBRANE, OPHTHALMOLOGICAL, | |
| OTHER AND UNSPECIFIED DRUGS AND MEDICAMENTS | |
| **EXCLUDED CATEGORIES** | |
| HANGING, STRANGULATION OR SUFFOCATION, UNDETERMINED WHETHER | |
| SUBMERSION (DROWNING), UNDETERMINED WHETHER ACCIDENTALLY OR PURPOSELY | |
| INJURY BY OTHER AND UNSPECIFIED MEANS, UNDETERMINED WHETHER | |
| LATE EFFECTS OF INJURY, UNDETERMINED WHETHER ACCIDENTALLY OR PURPOSELY | |
| LATE EFFECTS OF INJURY DUE TO WAR OPERATIONS | |
| HIT BY ROLLING STOCK | |
| RAILWAY ACCIDENT OF UNSPECIFIED NATURE | |
| ACCIDENT TO WATERCRAFT CAUSING SUBMERSION | |
| ACCIDENT TO WATERCRAFT CAUSING OTHER INJURY | |
| OTHER ACCIDENTAL SUBMERSION OR DROWNING IN WATER TRANSPORT ACCIDENT | |
| OTHER FALL FROM ONE LEVEL TO ANOTHER IN WATER TRANSPORT | |
| OTHER AND UNSPECIFIED FALL IN WATER TRANSPORT | |
| MACHINERY ACCIDENT IN WATER TRANSPORT | |
| OTHER AND UNSPECIFIED WATER TRANSPORT ACCIDENT | |
| ACCIDENT TO POWERED AIRCRAFT, OTHER AND UNSPECIFIED | |
| OTHER SPECIFIED AIR TRANSPORT ACCIDENTS | |
| ACCIDENTS INVOLVING POWERED VEHICLES USED SOLELY WITHIN THE BUILDINGS | |
| ACCIDENTS INVOLVING OTHER VEHICLES NOT ELSEWHERE CLASSIFIABLE | |
